# Association between patient safety culture, adverse events, and essential practices during childbirth in six Brazilian maternity hospitals

**DOI:** 10.1002/ijgo.70890

**Published:** 2026-03-12

**Authors:** Zenewton A. S. Gama, Cecília Olívia Paraguai de Oliveira Saraiva, Tatyana Maria Silva de Souza Rosendo, Rose L. Molina, Katherine E. A. Semrau, Danielle E. Tuller, Megan Marx Delaney, Christopher Westgard, Lauren Bobanski, Cláudia Maria Messias, Liana Carlan Padilha, Marise Reis de Freitas

**Affiliations:** ^1^ Department of Collective Health Federal University of Rio Grande do Norte–UFRN Natal Rio Grande do Norte Brazil; ^2^ Graduate Program in Quality Management in Health Services (QualiSaúde) Federal University of Rio Grande do Norte–UFRN Natal Rio Grande do Norte Brazil; ^3^ Ariadne Labs, Brigham and Women's Hospital and Harvard T. H. Chan School of Public Health Boston Massachusetts USA; ^4^ Department of Obstetrics and Gynecology Beth Israel Deaconess Medical Center Boston Massachusetts USA; ^5^ Department of Medicine Harvard Medical School Boston Massachusetts USA; ^6^ Division of Global Health Equity Brigham and Women's Hospital Boston USA; ^7^ Maternal, Child and Psychiatric Department Fluminense Federal University Rio de Janeiro Brazil; ^8^ Undergraduate Course in Collective Health Federal University of Rio Grande do Norte–UFRN Natal Rio Grande do Norte Brazil; ^9^ Department of Infectious Diseases Federal University of Rio Grande do Norte–UFRN Natal Rio Grande do Norte Brazil

**Keywords:** Brazil, maternal health, organizational culture, patient safety

## Abstract

**Objective:**

This study examines whether hospitals with stronger patient safety culture more consistently follow essential birth practices and have fewer adverse outcomes for mothers and newborns and whether this evidence can guide maternity care priorities in patient safety policy.

**Methods:**

We conducted a multicenter cross‐sectional study in six public maternity hospitals in Brazil (November 2022 to February 2024). In each hospital, we measured patient safety culture using the Brazilian version of the Hospital Survey on Patient Safety Culture. From a systematic sample of 2183 births (approximately 360 per hospital), we reviewed charts to record eight essential obstetric and neonatal practices and 10 adverse outcomes. We created hospital‐level composites for the practice bundle and adverse outcomes and correlated them with culture scores using Spearman coefficients (one‐sided exact *P*‐values).

**Results:**

A total of 686 professionals responded to the survey, with a mean overall culture score of 43.6% (range: 29.5%–56.4%). “Perception of safety” and “non‐punitive response to errors” were consistently low, while “management expectations,” “organizational learning,” and “teamwork within units” were relative strengths. Adherence was high for postpartum oxytocin (93.4%), vitamin K (95.9%), and newborn identification (91.1%), and low for partogram initiation (36.4%), birth companion (48.1%), and breastfeeding within the first hour (47.5%). Culture scores aligned positively with the practice bundle (*ρ =* 0.77; *P =* 0.072) and inversely with adverse outcomes—maternal (*ρ =* −0.77; *P =* 0.072), neonatal (*ρ =* −0.89; *P =* 0.019), and total (*ρ =* −0.94; *P =* 0.005).

**Conclusion:**

Findings support pairing culture‐strengthening actions with clinical bundles to promote safer childbirth and prioritize maternity services in safety policy.

## 1 INTRODUCTION

Global declines in maternal and neonatal mortality have plateaued, including in Brazil, indicating that expanding coverage alone is insufficient without systematic improvements in the quality and safety of childbirth care.^1^ Between 2000 and 2020, maternal deaths stagnated or rose in many regions.^2^ In Brazil, maternal mortality has remained above the Sustainable Development Goal target since 2015, and neonatal mortality continues to drive under‐5 deaths with marked regional inequities.^3^ These trends underscore the need to improve how health services organize and deliver care, not merely increase access.^4^

We treat patient safety culture (PSC—the shared values, beliefs, and behaviors that support safe practice—as a core organizational determinant of reliable care and reduced adverse events).^5^, ^6^ The World Health Organization (WHO) Global Patient Safety Action Plan 2021–2030 prioritizes PSC and calls for visible leadership, learning from events, and non‐punitive responses to error.^6^, ^7^ Maternity care exposes system fragilities because childbirth is time‐critical and unpredictable, involves a mother–newborn dyad, and requires coordination across professions, units, and settings.^8^

Evidence from different countries points to recurring PSC deficits in maternity services. In Brazil, weaker PSC has been associated with missed nursing care, with staffing and resource constraints as key drivers.^9^ Qualitative work in Portugal highlights communication failures, team instability, punitive climates, and under‐reporting of incidents,^10^ while Swedish studies show variation in PSC across professions and units, with teamwork explaining substantial variation in safety perceptions.^11^ Despite widespread use of validated instruments, most studies describe PSC levels or measurement properties; few link PSC to adherence to essential practices and maternal or neonatal adverse outcomes (AO), particularly in low‐ and middle‐income countries.^12^

To address this gap, we assessed patient safety culture in six Brazilian public maternity hospitals and examined hospital‐level associations between patient safety culture and (i) adherence to obstetric and neonatal essential practices and (ii) adverse outcomes occurrence. We hypothesized that hospitals with stronger PSC would show higher adherence to essential practices and lower adverse outcome indices.

## 2 METHODS

### 2.1 Study design and setting

We conducted a multicenter, cross‐sectional, ecological study at the hospital level in six public maternity hospitals in Rio Grande do Norte and Rio de Janeiro, Brazil, between November 2022 and February 2024. Patient safety culture was the exposure, measured once per hospital. Patients were not classified into exposure groups; all analyses were conducted at the hospital level, consistent with an ecological study design. Outcomes were hospital‐level indicators of adherence to eight evidence‐based essential practices and the occurrence of 10 maternal and neonatal adverse events, obtained via systematic chart review.

The network comprised two federally funded teaching hospitals (a university hospital and a public company hospital), two state hospitals providing care for standard‐risk pregnancies in smaller municipalities, one state referral hospital for high‐risk pregnancies in a state capital, and one large municipal high‐risk obstetric referral hospital in Rio de Janeiro. Catchment areas ranged from rural towns with fewer than 50 000 inhabitants to a major metropolitan capital with over six million inhabitants. Average monthly births ranged from 133 to 400, and all participating hospitals had fewer than 5000 births annually, reflecting the typical size of public maternity hospitals within the Brazilian Unified Health System. All hospitals belonged to the public health sector; private maternity facilities were not included. Each hospital reported designated personnel responsible for patient safety. Aggregate hospital characteristics are summarized in Table 1. Hospital risk profile and organizational characteristics were described for contextualization only; no stratification or case‐mix adjustment was performed. To preserve confidentiality, facilities were anonymized as H1–H6, and only aggregate data are presented.

**TABLE 1:** Characteristics of participating maternity hospitals.

Hospital	Management/ownership	Location (population)^a^	Risk profile^b^	Births/month (average)^c^
H1	State Health Secretariat (public)	Non‐capital municipality (≈81 000)	Standard risk	133
H2	Federal university teaching hospital (public)	Non‐capital municipality (≈37 000)	Standard risk	256
H3	Federal university teaching hospital (public)	State capital (≈751 000)	High‐risk referral	310
H4	State Health Secretariat (public)	Non‐capital municipality (≈47 000)	Standard risk	266
H5	State Health Secretariat (public)	State capital (≈751 000)	High‐risk referral	300
H6	Municipal Health Secretariat (public)	State capital (≈6 211 000)	High‐risk referral	400

*Note*: All maternity hospitals reported having personnel responsible for patient safety in their organizational unit.

^a^ Population: latest available municipal estimate.

^b^ High‐risk referral: routinely receives referrals for high‐risk obstetric care.

^c^ Births/month (average): monthly mean for the study period.

Data collection periods were aligned in four hospitals. In one hospital, the culture survey started immediately after the indicator window, while in another the indicator window began approximately 60 days after the survey. Because patient safety culture reflects relatively stable organizational norms and leadership practices over short periods, rather than short‐term performance fluctuations, and no major structural, staffing, or governance changes occurred during these intervals, this limited temporal misalignment is unlikely to meaningfully bias hospital‐level associations.

### 2.2 Study population and samples

#### 2.2.1 Patient safety culture (professionals)

We targeted approximately 100 respondents per hospital (approximately 600 overall), providing a maximum 10‐percentage‐point margin of error at 95% confidence for a proportion when *P* = 0.50. Eligible participants were higher education professionals and technicians directly involved in clinical care, unit management, or patient safety activities.

#### 2.2.2 Chart review (patients)

**FIGURE 1:**
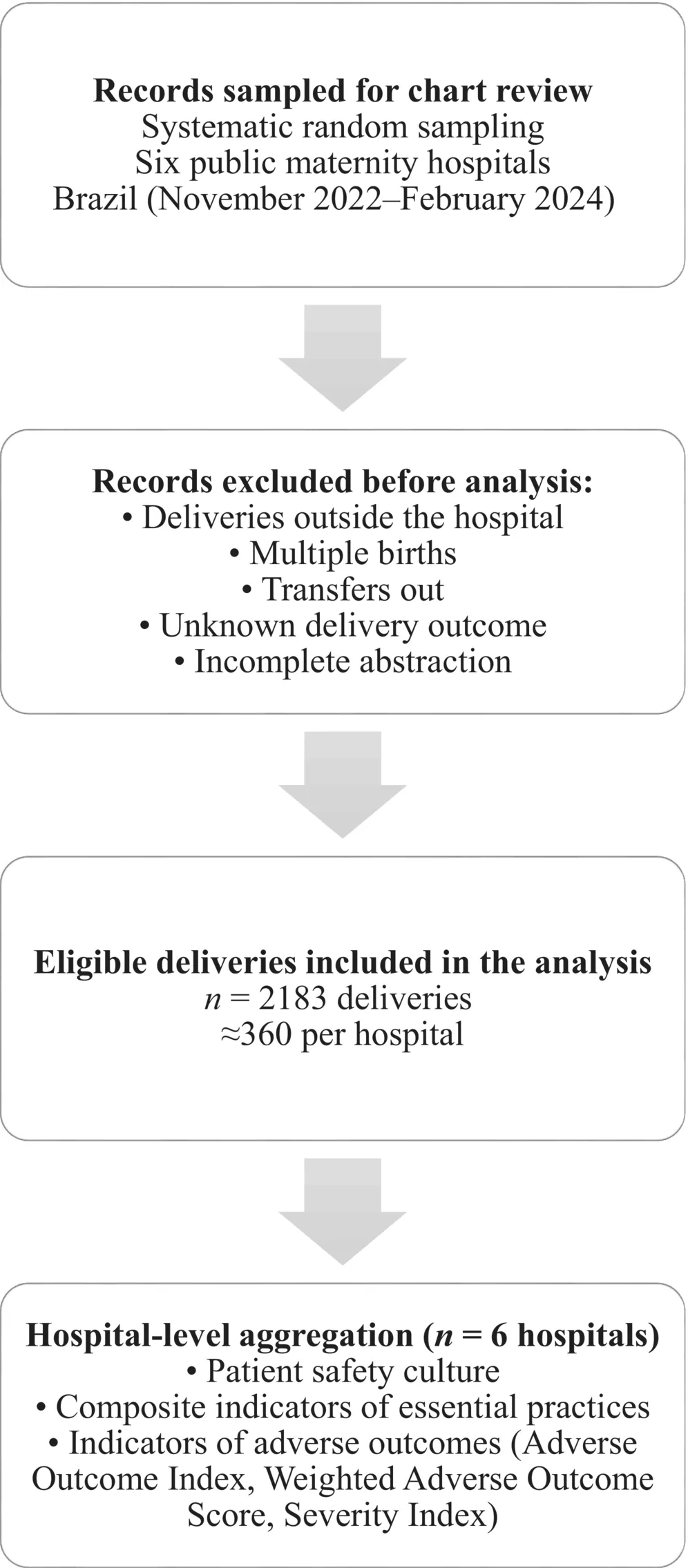
Flow diagram of chart review sampling and hospital‐level analysis.

For essential practices and adverse outcomes, we used systematic random sampling of 30 records every 2 weeks over 6 months per hospital (approximately 360 per hospital; planned total 2160; observed 2183 eligible encounters after cleaning). We included women admitted for delivery and their newborns ready for discharge. We excluded deliveries outside the hospital, multiple births, encounters with unknown delivery outcome, transfers out, and records with incomplete abstraction (i.e., documentation of only essential practices or only adverse outcomes). Figure 1 presents the flow diagram of record selection and exclusions.

### 2.3 Assessment of patient safety culture

We measured PSC using the Brazilian Hospital Survey on Patient Safety Culture E‐Questionnaire (60 items; 12 dimensions covering communication, teamwork, organizational learning, management support, and non‐punitive response to error), which also includes sociodemographic items.^13^ Following the instrument manual,^14^ we computed the percentage of positive responses overall and by dimension. “Agree/strongly agree” responses were coded as positive for agreement items and “most of the time/always” as positive for frequency items, after reverse‐coding negatively worded items. Dimension scores were calculated as the mean percentage of positive responses across items in that dimension; the overall score was the mean across all items (0%–100%, higher is better). Domains were classified as strengths when ≥75% of responses were positive and as weaknesses when <50% were positive.

In each hospital, the survey remained open for up to 90 days. Using systematic random sampling from staff rosters, local patient safety leads registered selected participants on the e‐platform. Invitation included study information, consent, and a questionnaire link; automated reminders were sent to non‐respondents. We also held in‐person sessions to facilitate smartphone completion.

### 2.4 Assessment of essential practices and adverse outcomes

We abstracted indicators from routinely maintained medical records using the QualiParto software (http://qualiparto.ccs.ufrn.br). Reviewers sampled 30 records every 2 weeks over a 6‐month period in each hospital, following a standardized abstraction protocol. We relied on routine documentation recorded by clinical teams during care delivery, which preserves ecological validity but might introduce variability in the completeness and detail of recorded information.

### 2.5 Essential practices (eight indicators)

Obstetric essential practices included partogram initiation; presence of a birth companion; oxytocin within 1 min of birth; and timely cord clamping, defined as delayed umbilical cord clamping documented as occurring at least 30–60 s after birth, regardless of mode of delivery. Neonatal essential practices: skin‐to‐skin within 1 h; breastfeeding within 1 h; vitamin K administration; newborn wristband identification.

Essential practices were assessed exclusively based on routine medical record documentation. Information on patient refusal of specific practices or preference for physiological management of the third stage of labor was not systematically recorded in the medical charts and, therefore, could not be captured or analyzed in this study.

For each hospital, adherence to each practice was calculated as the proportion of eligible deliveries in which the practice was documented as performed. Composite adherence indicators were computed at the hospital level as the unweighted mean of individual practice adherence, overall and by obstetric and neonatal domains.

### 2.6 Adverse outcomes (10 indicators)

Maternal adverse outcomes (*n* = 6) included maternal death; uterine rupture; return to surgery; third/fourth‐degree perineal laceration; blood transfusion; intensive care unit (ICU) admission. Neonatal adverse outcomes (*n* = 4) included birth trauma, defined as any documented neonatal injury recorded in the medical chart; neonatal ICU admission >24 h among newborns ≥2500 g; neonatal death; 5‐min Apgar <7.

We summarized composite adverse outcomes using established indices^15^: the adverse outcome index (AOI; proportion of deliveries with ≥1 adverse event), the weighted adverse outcome score (WAOS; severity‐weighted score per delivery), and the severity index (SI; severity‐weighted score per delivery among those with ≥1 event). Table S1 details weights and scoring.

### 2.7 Data management and analysis

We analyzed data at the hospital level. For each hospital, we summarized PSC (overall and by domain), composite adherence to essential practices (overall and obstetric/neonatal), and composite adverse outcomes (maternal, neonatal, and total AOI, plus descriptive WAOS and SI). We reported counts and proportions per hospital and overall.

To test whether hospitals with more favorable culture had better practices and fewer adverse outcomes, we estimated the Spearman rank correlation (*ρ*) between culture measures (overall and domains) and composite practice and outcome indicators, using one‐sided exact *P*‐values with a significance threshold of 0.05 (directional: hypotheses: *ρ* > 0 for essential practices; *ρ* > 0 for adverse outcomes).

One‐sided tests were pre‐specified based on clear directional hypotheses grounded in patient safety and quality‐of‐care theory, whereby stronger patient safety culture was expected to be associated with higher adherence to essential practices and lower rates of adverse outcomes; associations in the opposite direction were not of interest. Given the exploratory nature of this ecological, cross‐sectional analysis and small number of hospitals, one‐sided tests were used to improve statistical power to detect associations in the hypothesized direction.

Correlations were interpreted as exploratory and non‐causal. Prior to analysis, duplicate records were removed; multiple births, records with incomplete abstraction, and encounters with inconsistent admission or discharge dates were excluded. Analyses were conducted using IBM SPSS Statistics, version 29 (IBM, Armonk, NY, USA). No formal sensitivity analyses were performed.

### 2.8 Ethics

The Research Ethics Committee of Onofre Lopes University Hospital (CAAE 61091022.6.1001.5292) approved the study. Although this hospital was not a study site, its ethics committee serves as a regional reference board and reviewed the protocol in accordance with national regulations. All participating hospitals provided written authorization and were registered as cooperating institutions in Plataforma Brasil. We exported only de‐identified analytic variables and hospital‐level aggregates; no patient‐identifiable information or linkage keys left clinical sites. Survey invitations were managed locally, and the analytic dataset contained no names, emails, medical record numbers, full dates of birth, or addresses.

## 3 RESULTS

### 3.1 Participant characteristics

We surveyed 686 professionals across the six maternity hospitals (H1 = 126; H2 = 122; H3 = 32; H4 = 251; H5 = 140; H6 = 15). Most respondents worked on the clinical frontline, predominantly in nursing (43.3%; 297/686), and 63.8% (438/686) reported direct patient contact. Maternity care in Brazil is predominantly delivered by physicians and obstetric nurses rather than midwives; survey respondents reflected this professional mix. Nearly half (48.1%; 330/686) had ≥5 years in the service. The full professional breakdown by category and hospital is provided in Table S2.

### 3.2 Patient safety culture

Overall, PSC was low (mean 43.6%). Three domains reached ≥50% positive responses in most sites: D3 (supervisor/manager actions supporting safety), D4 (organizational learning/continuous improvement), and D5 (teamwork within unit). In contrast, D2 (safety perception) and D8 (non‐punitive response to errors) remained <50% in all hospitals. Only H2 (51.1%) and H4 (56.4%) exceeded 50% in the total PSC score. Domain‐level and overall PSC scores by hospital are shown in Table 2.

**TABLE 2:** Percentage of positive responses by patient safety culture dimension and maternity hospital, six Brazilian institutions, 2024.

Patient safety culture dimension	H1% (*n* = 126)	H2% (*n* = 122)	H3% (*n* = 32)	H4% (*n* = 251)	H5% (*n* = 140)	H6% (*n* = 15^d^ ^)^	Overall (all hospitals, % positive)^a^, ^c^
D1—Frequency of event reporting	**30.0**	**44.6**	**22.8**	53.1	**41.8**	**42.9**	**42.4**
D2—Safety perception	**30.7**	**43.0**	**25.0**	**40.9**	**29.6**	**30.5**	**30.6**
D3—Supervisor/Manager support for safety	73.7	70.7	57.0	84.7	74.0	60.0	72.2
D4—Organizational learning —continuous improvement	68.8	79.2	75.8	79.9	70.1	63.6	73.0
D5—Teamwork within the unit	56.6	80.2	58.6	69.9	60.3	**49.2**	59.5
D6—Openness to communication	**42.1**	52.1	**30.1**	51.1	**47.3**	**34.1**	**44.7**
D7—Feedback and communication about errors	**34.7**	50.0	**28.0**	55.2	**45.3**	**41.9**	**43.6**
D8—Non‐punitive response to errors	**18.6**	**27.5**	**14.7**	**26.8**	**22.8**	**20.9**	**21.9**
D9—Adequate staffing levels	52.3	**44.7**	**22.7**	52.3	**42.5**	**38.3**	**43.6**
D10—Hospital leadership support for safety	**43.8**	60.9	**28.8**	62.9	**42.6**	**43.6**	**43.7**
D11—Cross‐unit teamwork	**43.9**	59.3	**37.4**	61.2	**39.2**	**49.0**	**46.5**
D12—Handoffs and transitions between units	**45.2**	**49.0**	**41.9**	57.6	**40.3**	52.0	**47.1**
TOTAL—Overall PSC score^b^	**43.9**	51.1	**29.5**	56.4	**42.6**	**43.3**	**43.6**

*Note*: H1–H6 denote the six anonymized participating maternity hospitals. Bold indicates <50% positive responses (lower PSC).

^a^ “Overall” column shows the all‐hospital average for each dimension (unweighted by site size).

^b^ Overall PSC score (TOTAL): unweighted mean of the 12 dimension‐level % positives.

^c^ Positive response defined per HSOPS: “Agree/Strongly agree” or “Most of the time/Always”; reverse‐coded items recoded; dimension computed when ≥50% of items answered.

^d^ Small sample: estimates for H6 (*n* = 15) should be interpreted with caution.

### 3.3 Essential practices and adverse events

Adherence was high for postpartum oxytocin (93.4%), vitamin K (95.9%), and newborn identification (91.1%), and low for partogram initiation (36.4%), early breastfeeding within 1 h (47.5%), and presence of a birth companion (48.1%), with marked between‐hospital variation (e.g., companion presence 0.8% in H1 vs. 99.7% in H2). For the overall essential practices composite, only H2 reached ≥80% (80.7%); H4 and H1 followed, with 74.3% and 73.4%, respectively (Table 3).

**TABLE 3:** Indicators of essential practices (EP) for maternal and newborn care in six Brazilian maternity hospitals, 2024.

Essential Practices	H1 Deliveries observed: *N* = 360^a^	H2 Deliveries observed: *N* = 360^a^	H3 Deliveries observed: *N* = 344^a^	H4 Deliveries observed: *N* = 360^a^	H5 Deliveries observed: *N* = 383^a^	H6 Deliveries observed: *N* = 376^a^	Total *N* = 2183^a^
Mother							
Oxytocin administred	327 (90.8)	352 (97.8)	317 (92.2)	348 (96.7)	343 (89.6)	352 (93.6)	2040 (93.4)
Presence of a Companion^b^	**3 (0.8)** ^b^	359 (99.7)	266 (77.3)	231 (64.2)	**25 (6.5)** ^b^	**167 (44.4)**	**1051 (48.1)**
Partogram initiated	**187 (51.9)**	**152 (42.2)**	**113 (32.8)**	226 (62.8)	**0 (0.0)** ^d^	**117 (31.1)**	**795 (36.4)**
Composite (maternal) (%)	**56.9**	80.2	72	84.6	**34.4**	**58.2**	64
Newborn							
Vitamin K administered	357 (99.2)	358 (99.4)	280 (81.4)	358 (99.4)	378 (98.7)	364 (96.8)	2095 (95.9)
Newborn wristband identification	353 (98.1)	254 (70.6)	280 (81.4)	327 (90.8)	364 (95.0)	342 (91.0)	1920 (91.1)
Timely umbilical cord clamping	235 (65.3)	307 (85.3)	254 (73.8)	**124 (34.4)**	266 (69.5)	**164 (43.6)**	1350 (64.1)
Skin‐to‐skin contact initiated	293 (81.4)	293 (81.4)	**139 (40.4)**	**119 (33.1)**	233 (60.8)	**157 (41.8)**	**1234 (58.6)**
Early postpartum breastfeeding initiated	**182 (50.6)**	244 (67.8)	**129 (37.5)**	**77 (21.4)**	**130 (33.9)**	238 (63.3)	**1000 (47.5)**
Composite (newborn) (%)	82.1	80.9	60.2	67.8	81.5	**53.2**	73.3
Overall composite EP (%)^c^	73.4	80.7	64.3	74.3	63.1	64.2	69.9

*Notes*: H1–H6 denote the six anonymized participating maternity hospitals. Cell values are *n* (%); denominators reflect eligible births per indicator (eligibility and exclusions in Methods). Bold indicates adherence <60%.

^a^ Hospital headers show total deliveries observed during the period (*N*).

^b^ Companion presence restricted and/or documentation issues during the period.

^c^ Composite EP is the unweighted mean of indicator‐level adherence at the hospital level (reported for maternal, newborn, and overall).

^d^ Hospital did not routinely use the partograph during the period; value reflects adherence as recorded (see Methods).

The most frequent AE was neonatal ICU admission >24 h among newborns ≥2500 g (2.0%; 43/2183), concentrated in H5 (3.7%) and H3 (3.8%). Severe adverse outcomes included 37 neonatal deaths (1.7%) across all hospitals (higher in H5 and H6) and 33 maternal ICU admissions (1.5%), notably in H3 (7.3%). We observed six maternal deaths (0.3%) and three uterine ruptures (0.1%).

Non‐risk‐adjusted composites showed higher total AOI in H3 (7.6%) and H5 (6.0%), with lower values in H2 (1.7%) and H4 (1.8%). WAOS was highest in H5 (30.7) and H6 (27.3); and SI was highest in H6 (340.6) and H5 (273.5) (Table 4).

**TABLE 4:** Indicators of maternal and neonatal adverse events in six Brazilian maternity hospitals, 2024.

Adverse events	H1 deliveries observed: *N* = 360	H2 deliveries observed: *N* = 360	H3 deliveries observed: *N* = 344	H4 deliveries observed: *N* = 360	H5 deliveries observed: *N* = 383	H6 deliveries observed: *N* = 376	Total *N* = 2183
Mother							
Return to delivery room or surgery	5 (1.4)	1 (0.3)	**9 (2.6)**	5 (1.4)	**7 (1.8)**	**9 (2.4)**	36 (1.6)
Blood transfusion received	**3 (0.8)**	0 (0.0)	5 (1.5)	**4 (1.1)**	**3 (0.8)**	0 (0.0)	15 (0.7)
Admitted to ICU	2 (0.6)	0 (0.0)	**25 (7.3)**	0 (0.0)	5 (1.3)	1 (0.3)	33 (1.5)
3rd–4th degree perineal laceration	**3 (0.8)**	**3 (0.8)**	1 (0.3)	**4 (1.1)**	1 (0.3)	2 (0.5)	14 (0.6)
Maternal death	0 (0.0)	0 (0.0)	**2 (0.6)**	1 (0.3)	**3 (0.8)**	0 (0.0)	6 (0.3)
Uterine rupture	**2 (0.6)**	0 (0.0)	0 (0.0)	0 (0.0)	**1 (0.3)**	0 (0.0)	3 (0.1)
Composite AOI (maternal) %	**4.7**	1.1	**10.8**	3.1	3.7	3.2	4.4
Newborn							
NICU admission >24 h among newborns >2500 g	2 (0.6)	5 (1.4)	**13 (3.8)**	0 (0.0)	**14 (3.7)**	**9 (2.4)**	43 (2.0)
Neonatal death	1 (0.3)	1 (0.3)	2 (0.6)	2 (0.6)	**20 (5.2)**	**11 (2.9)**	37 (1.7)
Apgar <7 in at 5th min	2 (0.6)	2 (0.6)	0 (0.0)	2 (0.6)	**10 (2.6)**	**11 (2.9)**	27 (1.2)
Birth‐related trauma or injury	**2 (0.6)**	0 (0.0)	**1 (0.3)**	0 (0.0)	0 (0.0)	0 (0.0)	3 (0.1)
Composite AOI (newborn) %	1.1	2.2	**4.4**	0.6	**8.4**	3.2	3.4
Composite measures							
AOI Mother + Newborn (%)	2.9	1.7	7.6	1.8	6.0	3.2	7.8
WAOS Mother + Newborn	3.5	1.9	**14.3**	5.3	**30.7**	**27.3**	13.6
SI Mother + Newborn	73.2	56.7	104.4	146.2	**273.5**	**340.6**	183.8

*Note*: H1–H6 denote the six anonymized participating maternity hospitals. Cell values are *n* (%) based on all observed deliveries unless clinical ineligibility/exclusions are specified in Section 2. Hospital headers show total deliveries observed. Bold indicates rates worse than the overall (Total) for that row. AOI (adverse outcome index) Mother + Newborn: hospital‐level overall adverse outcome index (% of deliveries with ≥1 included adverse event). WAOS (weighted adverse outcome score): weighted score reflecting frequency and severity of adverse outcomes. Severity index (SI): WAOS divided by AOI (higher values indicate greater severity per adverse‐affected delivery).

### 3.4 Correlation between patient safety culture and performance indicators

At the hospital level (*n* = 6), higher PSC generally aligned with better care processes and fewer AOs: PSC total correlated positively with total essential practices and negatively with maternal, neonatal, and total AOI, with moderate‐to‐large magnitudes. Using directional, one‐sided exact tests, two associations met nominal significance: PSC with neonatal AOI and with total AOI (*ρ* negative; *P* < 0.05). Associations with maternal AOI and total essential practices were borderline (*p* = 0.05).

**FIGURE 2:**
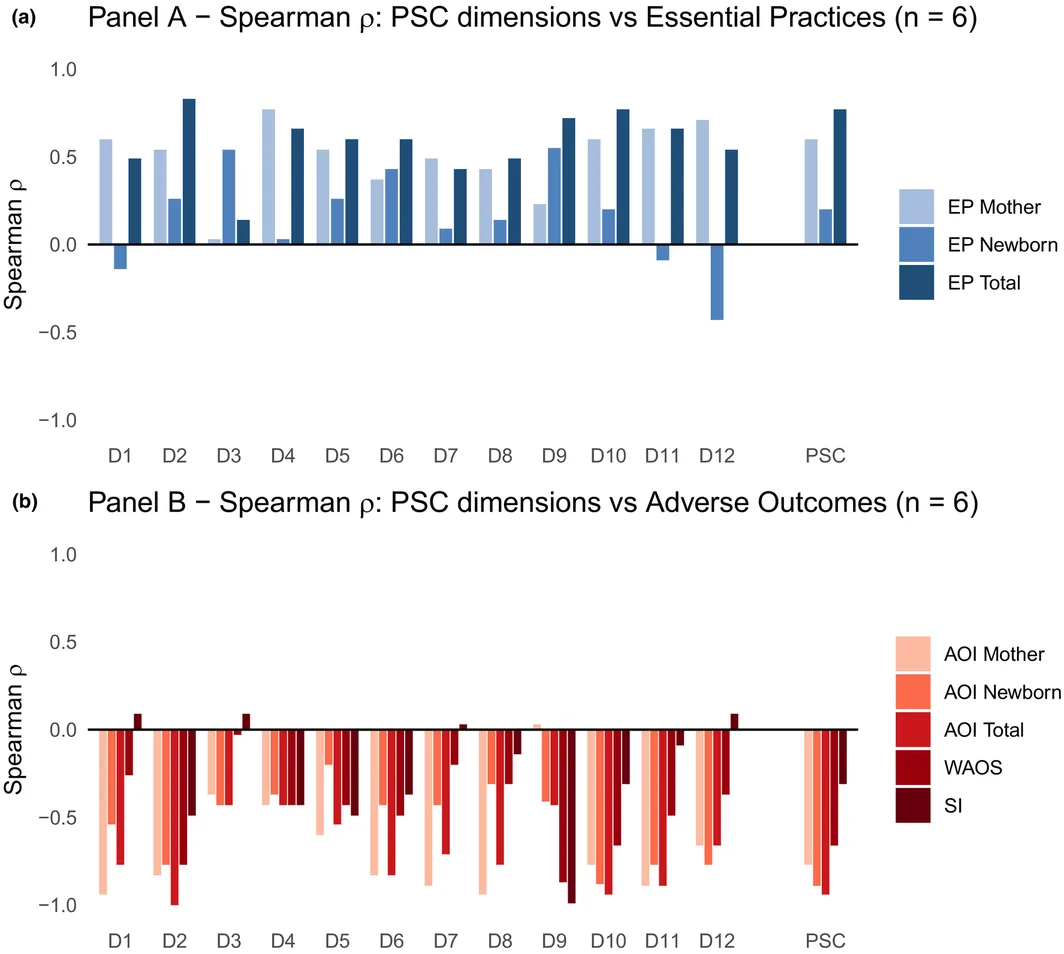
Hospital‐level Spearman correlations (ρ) between patient safety culture (PSC; domains D1–D12 and PSC Total) and (a) essential practices (EP: Maternal, newborn, total) and (b) adverse outcomes (AOI: Maternal, newborn, total; WAOS; Severity Index). Bars show ρ on a common scale (−1 to +1) across panels; higher values in Panel A indicate higher PSC associated with higher EP, and more negative values in Panel B indicate higher PSC associated with lower adverse outcomes. Analyses are exploratory and unadjusted. No significance markers are shown in the figure. Exact correlation coefficients and one‐sided exact *P*‐values (directional: EP *ρ* > 0; adverse outcomes *ρ* < 0) are provided in Table S3. Results reflect hospital‐level associations (*n* = 6; Spearman rank correlation.

At the domain level, patterns broadly mirrored PSC total. Essential practices showed the strongest positive associations with D2 (safety perception) and D10 (leadership support) (*P* < 0.05). For total AOI, inverse associations were most evident for D2 (safety perception), D6 (open communication), D10 (leadership support), and D11 (cross‐unit teamwork) (*P* < 0.05). These exploratory correlations are summarized in Figure 2 and Table S3.

## 4 DISCUSSION

### 4.1 Principal findings

Across six Brazilian maternity hospitals, PSC was generally low. Hospitals with stronger PSC tended to have higher adherence to essential practices and lower maternal, newborn, and total adverse outcomes. Safety perception and non‐punitive response to errors were consistently weak, whereas unit management expectations/actions, organizational learning, and teamwork within units were comparatively stronger.

### 4.2 Interpretation in the Brazilian policy context

WHO emphasizes strengthening PSC, including in maternity care; World Patient Safety Day 2021 highlighted reporting and learning from incidents, avoiding unnecessary practices, workforce capability, and safer use of medications and transfusions during childbirth.^16^ Measuring PSC helps reveal organizational conditions that shape outcomes, but it requires local engagement and evaluation capacity.^17^ We theorize that stronger safety culture—especially non‐punitive climate, managerial support, and teamwork—enables more reliable coordination and handoffs, thereby improving essential practices adherence and reducing adverse outcomes. Safety perception may behave partly as a climate barometer, responsive to recent performance, whereas leadership support has clearer pathways to improved coordination and practice reliability.

In Brazil, the maternal–newborn safety agenda has largely centered on epidemiological surveillance at the Ministry of Health, which treats maternal death as a sentinel event and convenes review committees.^18^ This approach targets clinical causes (hypertension, infection, hemorrhage, and unsafe abortion) but pays less attention to organizational determinants such as teamwork, leadership, organizational learning, and open communication. In parallel, the National Patient Safety Program, launched in 2013 under sanitary surveillance (ANVISA), initially prioritized services such as ICUs and dialysis; maternity services were not early targets.^19^, ^20^, ^21^

Our findings are compatible with a plausible hypothesis—without implying causality—that this programmatic split (epidemiological surveillance leading the maternal agenda and sanitary surveillance leading patient safety elsewhere) might have slowed the uptake of safety practices in maternity hospitals. PSC levels in our sample appear lower than national hospitals averages,^22^ suggesting that patient safety remains secondary to clinically driven childbirth agendas.

Concretely, Brazil could (i) incorporate PSC measurement and event‐learning processes into routine maternal–neonatal surveillance; (ii) make leadership accountability for safety explicit at maternity‐service level (rounds, huddles, debriefs, non‐punitive reporting); and (iii) pair essential clinical bundles with culture‐focused interventions (cross‐unit teamwork, reliable handoffs, and feedback loops). Organizational factors, not only clinical bundles, are necessary to sustain safer childbirth care.

### 4.3 Comparison with the literature

Low PSC in our sample aligns with studies from Malaysia, the AHRQ database, and Portuguese maternity units, where overall scores also fell below the “strong” threshold (>75%).^10^, ^23^, ^24^ Safety perception and non‐punitive response to errors emerge as recurring deficits; the latter is consistently described as central to learning cultures.^10^, ^25^, ^26^ Brazil's national data show a similar pattern, with safety perception and non‐punitive response as weaknesses and unit management and organizational learning as strengths.^22^ On domain‐level correlations—linking safety perception, open communication, leadership support, and cross‐unit teamwork to fewer adverse outcomes—echo international evidence connecting managerial support and non‐punitive climates to learning and reliability.

### 4.4 Strengths and limitations

Strengths of this study include its multi‐hospital design; the integration of PSC, care process, and outcome indicators; and the focus on public‐sector maternity care in an upper‐middle‐income setting. Key limitations include the hospital‐level unit of analysis with a small number of hospitals (*n* = 6), the cross‐sectional design, a small PSC sample in one hospital (*n* = 15), the absence of adjustment for multiple testing, potential case‐mix differences across facilities, and reliance on self‐report culture measures and documentation‐based assessment of essential practices and adverse events.

Given the limited sample size and the examination of multiple correlated domains and outcomes, findings should be interpreted cautiously, particularly those with marginal statistical significance. In addition, Brazil has one of the highest cesarean section rates worldwide, which might influence both care processes and adverse outcome rates; however, mode of delivery was not a focus of the present analysis and was not used for stratification. These factors underscore the exploratory nature of the findings and motivate confirmatory studies with larger samples, risk adjustment, and prospective designs.

### 4.5 Implications for practice, policy, and research

Our data suggest that organizational conditions matter for the reliability of childbirth care; that is, for the consistent delivery of essential practices. Implementation efforts such as the WHO Safe Childbirth Checklist and WHO Labor Care Guide, as well as structured shared decision‐making approaches (e.g., team birth), might benefit from measuring PSC at baseline, addressing cultural gaps (non‐punitive climate, leadership support, cross‐unit coordination), and tracking culture and processes alongside outcomes. When comparing sites or evaluating impact, PSC should be considered as a covariate, effect modifier, or mediator.

Prospective studies should test whether culture‐oriented strategies (e.g., leadership walk rounds, safety huddles, structured debriefs, non‐punitive reporting and feedback, and standardized handoffs) improve essential practices adherence and reduce adverse outcomes. Suitable designs include interrupted time series and cluster trials with multilevel models. Future work should also compare PSC across facility types using stratified sampling and tests of measurement invariance and strengthen essential practices measurement by combining routine records with direct observation or validated chart‐audit protocols. Replication across diverse low‐ and middle‐income country settings, with linkage to mortality and severe‐morbidity registries, would enhance external validity.

## 5 CONCLUSION

Across six Brazilian maternity hospitals, PSC was generally low. At the hospital level, stronger culture aligned with higher adherence to essential practices and lower adverse event indices. Adherence varied widely (high for postpartum oxytocin, vitamin K, and newborn identification and low for partogram initiation, companion presence, and early breastfeeding), indicating organization‐dependent gaps. These observational, unadjusted findings suggest that strengthening organizational conditions (e.g., through leadership support for patient safety, cross‐unit teamwork and reliable handoffs, open communication and feedback, and a non‐punitive climate) might support safer childbirth care. Given the design and small sample, we urge caution and recommend prioritizing maternity services within patient‐safety policy and prospectively testing culture‐strengthening strategies alongside clinical bundles.

## AUTHOR CONTRIBUTIONS

Each co‐author contributed to the research and the drafting of the manuscript, as detailed below: Zenewton André da Silva Gama: Contributed to the conception and design of the work; analysis and interpretation of data; drafting the work and reviewing it critically, providing important intellectual content; final approval of the manuscript's version to be published. The author agrees to be accountable for all aspects of the work in ensuring that questions related to the accuracy or integrity of any part of the work are appropriately investigated and resolved. Cecília Olívia Paraguai de Oliveira Saraiva: Contributed to the conception and design of the work; acquisition, analysis, and interpretation of data; drafting the manuscript and critically revising it for important intellectual content; and final approval of the version to be published. The author agrees to be accountable for all aspects of the work, ensuring that any questions related to the accuracy or integrity of any part are appropriately investigated and resolved. Tatyana Maria Silva de Souza Rosendo: Contributed to the conception and design of the work; acquisition, analysis, and interpretation of data; drafting the manuscript and critically revising it for important intellectual content; and final approval of the version to be published. The author agrees to be accountable for all aspects of the work, ensuring that any questions related to the accuracy or integrity of any part are appropriately investigated and resolved. Rose L. Molina: Contributed to the analysis, and interpretation of data; drafting the manuscript and critically revising it for important intellectual content; and final approval of the version to be published. The author agrees to be accountable for all aspects of the work, ensuring that any questions related to the accuracy or integrity of any part are appropriately investigated and resolved. Katherine E. A. Semrau: contributed to the analysis, and interpretation of data; drafting the manuscript and critically revising it for important intellectual content; and final approval of the version to be published. The author agrees to be accountable for all aspects of the work, ensuring that any questions related to the accuracy or integrity of any part are appropriately investigated and resolved. Danielle E. Tuller: Contributed to the analysis, and interpretation of data; drafting the manuscript and critically revising it for important intellectual content; and final approval of the version to be published. The author agrees to be accountable for all aspects of the work, ensuring that any questions related to the accuracy or integrity of any part are appropriately investigated and resolved. Megan Marx Delaney: Contributed to the analysis, and interpretation of data; drafting the manuscript and critically revising it for important intellectual content; and final approval of the version to be published. The author agrees to be accountable for all aspects of the work, ensuring that any questions related to the accuracy or integrity of any part are appropriately investigated and resolved. Christopher Westgard: Contributed to the analysis, and interpretation of data; drafting the manuscript and critically revising it for important intellectual content; and final approval of the version to be published. The author agrees to be accountable for all aspects of the work, ensuring that any questions related to the accuracy or integrity of any part are appropriately investigated and resolved. Lauren Bobanski: Contributed to the analysis, and interpretation of data; drafting the manuscript and critically revising it for important intellectual content; and final approval of the version to be published. The author agrees to be accountable for all aspects of the work, ensuring that any questions related to the accuracy or integrity of any part are appropriately investigated and resolved. Cláudia Maria Messias: Contributed to the acquisition, analysis, and interpretation of data; drafting the manuscript and critically revising it for important intellectual content; and final approval of the version to be published. The author agrees to be accountable for all aspects of the work, ensuring that any questions related to the accuracy or integrity of any part are appropriately investigated and resolved. Liana Carlan Padilha: Contributed to the acquisition, analysis, and interpretation of data; drafting the manuscript and critically revising it for important intellectual content; and final approval of the version to be published. The author agrees to be accountable for all aspects of the work, ensuring that any questions related to the accuracy or integrity of any part are appropriately investigated and resolved. Marise Reis de Freitas: Contributed to the analysis, and interpretation of data; drafting the manuscript and critically revising it for important intellectual content; and final approval of the version to be published. The author agrees to be accountable for all aspects of the work, ensuring that any questions related to the accuracy or integrity of any part are appropriately investigated and resolved.

## FUNDING INFORMATION

The study was funded by the Brazilian National Council for Scientific and Technological Development (CNPq), process number 409972/2021‐5, under a Universal Call for Projects grant.

## CONFLICT OF INTEREST STATEMENT

The authors declare that they have no conflicts of interest related to this study.

## Supporting information


Table S1.

Table S2.

Table S3.

## Data Availability

Research data are not shared.
