# Supplementary material for: Association between patient safety culture, adverse events, and essential practices during childbirth in six Brazilian maternity hospitals
Source: Int J Gynaecol Obstet. 2026 Mar 12;174(3):1316–27. doi: 10.1002/ijgo.70890 (PMC13505257; doi:10.1002/ijgo.70890)
Supplement: Supplementary file 1 [file IJGO-174-1316-s001.docx]

**Supplementary Table S3.**

**Hospital-level Spearman correlations (ρ) between patient safety culture (PSC; domains D1-D12 and PSC Total) and process/outcome indicators, with directional one-sided exact *p*-values.**

For Essential Practices (EP) outcomes (EP_Mother, EP_Newborn, EP_Total) we tested H1: ρ > 0; for adverse outcomes (AOI_Mother, AOI_Newborn, AOI_Total, WAOS_Total, SI_Total) we tested H1: ρ < 0. With n = 6 hospitals, exact one-sided p-values were obtained from the permutation distribution of Spearman’s ρ by enumerating all 6! permutations of ranks and computing the tail probability in the hypothesized direction. Analyses are exploratory; no multiplicity adjustments were applied.

| **Outcome** | **PSC_dimension** | **Rho** | **p_one_sided_exact** | **n_pairs** | **Hypothesis** |
| --- | --- | --- | --- | --- | --- |
| EP_Mother | D1 | 0,6 | 0.121 | 6 | r>0 (EP) |
| EP_Mother | D2 | 0,54 | 0.149 | 6 | r>0 (EP) |
| EP_Mother | D3 | 0,03 | 0.460 | 6 | r>0 (EP) |
| EP_Mother | D4 | 0,77 | 0.051 | 6 | r>0 (EP) |
| EP_Mother | D5 | 0,54 | 0.149 | 6 | r>0 (EP) |
| EP_Mother | D6 | 0,37 | 0.249 | 6 | r>0 (EP) |
| EP_Mother | D7 | 0,49 | 0.149 | 6 | r>0 (EP) |
| EP_Mother | D8 | 0,43 | 0.178 | 6 | r>0 (EP) |
| EP_Mother | D9 | 0,23 | 0.329 | 6 | r>0 (EP) |
| EP_Mother | D10 | 0,6 | 0.121 | 6 | r>0 (EP) |
| EP_Mother | D11 | 0,66 | 0.068 | 6 | r>0 (EP) |
| EP_Mother | D12 | 0,71 | 0.068 | 6 | r>0 (EP) |
| EP_Mother | PSC | 0,6 | 0.121 | 6 | r>0 (EP) |
| EP_Newborn | D1 | -0,14 | 0.599 | 6 | r>0 (EP) |
| EP_Newborn | D2 | 0,26 | 0.282 | 6 | r>0 (EP) |
| EP_Newborn | D3 | 0,54 | 0.149 | 6 | r>0 (EP) |
| EP_Newborn | D4 | 0,03 | 0.460 | 6 | r>0 (EP) |
| EP_Newborn | D5 | 0,26 | 0.282 | 6 | r>0 (EP) |
| EP_Newborn | D6 | 0,43 | 0.178 | 6 | r>0 (EP) |
| EP_Newborn | D7 | 0,09 | 0.401 | 6 | r>0 (EP) |
| EP_Newborn | D8 | 0,14 | 0.401 | 6 | r>0 (EP) |
| EP_Newborn | D9 | 0,55 | 0.121 | 6 | r>0 (EP) |
| EP_Newborn | D10 | 0,2 | 0.357 | 6 | r>0 (EP) |
| EP_Newborn | D11 | -0,09 | 0.599 | 6 | r>0 (EP) |
| EP_Newborn | D12 | -0,43 | 0.822 | 6 | r>0 (EP) |
| EP_Newborn | PSC | 0,2 | 0.357 | 6 | r>0 (EP) |
| EP_Total | D1 | 0,49 | 0.149 | 6 | r>0 (EP) |
| EP_Total | D2 | 0,83 | 0.017* | 6 | r>0 (EP) |
| EP_Total | D3 | 0,14 | 0.401 | 6 | r>0 (EP) |
| EP_Total | D4 | 0,66 | 0.068 | 6 | r>0 (EP) |
| EP_Total | D5 | 0,6 | 0.121 | 6 | r>0 (EP) |
| EP_Total | D6 | 0,6 | 0.121 | 6 | r>0 (EP) |
| EP_Total | D7 | 0,43 | 0.178 | 6 | r>0 (EP) |
| EP_Total | D8 | 0,49 | 0.149 | 6 | r>0 (EP) |
| EP_Total | D9 | 0,72 | 0.051 | 6 | r>0 (EP) |
| EP_Total | D10 | 0,77 | 0.051 | 6 | r>0 (EP) |
| EP_Total | D11 | 0,66 | 0.068 | 6 | r>0 (EP) |
| EP_Total | D12 | 0,54 | 0.149 | 6 | r>0 (EP) |
| EP_Total | PSC | 0,77 | 0.051 | 6 | r>0 (EP) |
| AOI_Mother | D1 | -0,94 | 0.008* | 6 | r<0 (Adverse) |
| AOI_Mother | D2 | -0,83 | 0.017* | 6 | r<0 (Adverse) |
| AOI_Mother | D3 | -0,37 | 0.249 | 6 | r<0 (Adverse) |
| AOI_Mother | D4 | -0,43 | 0.178 | 6 | r<0 (Adverse) |
| AOI_Mother | D5 | -0,6 | 0.121 | 6 | r<0 (Adverse) |
| AOI_Mother | D6 | -0,83 | 0.017* | 6 | r<0 (Adverse) |
| AOI_Mother | D7 | -0,89 | 0.008* | 6 | r<0 (Adverse) |
| AOI_Mother | D8 | -0,94 | 0.008 | 6 | r<0 (Adverse) |
| AOI_Mother | D9 | 0,03 | 0.540 | 6 | r<0 (Adverse) |
| AOI_Mother | D10 | -0,77 | 0.051 | 6 | r<0 (Adverse) |
| AOI_Mother | D11 | -0,89 | 0.008 | 6 | r<0 (Adverse) |
| AOI_Mother | D12 | -0,66 | 0.068 | 6 | r<0 (Adverse) |
| AOI_Mother | PSC | -0,77 | 0.051 | 6 | r<0 (Adverse) |
| AOI_Newborn | D1 | -0,54 | 0.149 | 6 | r<0 (Adverse) |
| AOI_Newborn | D2 | -0,77 | 0.051 | 6 | r<0 (Adverse) |
| AOI_Newborn | D3 | -0,43 | 0.178 | 6 | r<0 (Adverse) |
| AOI_Newborn | D4 | -0,37 | 0.249 | 6 | r<0 (Adverse) |
| AOI_Newborn | D5 | -0,2 | 0.357 | 6 | r<0 (Adverse) |
| AOI_Newborn | D6 | -0,43 | 0.178 | 6 | r<0 (Adverse) |
| AOI_Newborn | D7 | -0,43 | 0.178 | 6 | r<0 (Adverse) |
| AOI_Newborn | D8 | -0,31 | 0.282 | 6 | r<0 (Adverse) |
| AOI_Newborn | D9 | -0,41 | 0.210 | 6 | r<0 (Adverse) |
| AOI_Newborn | D10 | -0,88 | 0.017* | 6 | r<0 (Adverse) |
| AOI_Newborn | D11 | -0,77 | 0.051 | 6 | r<0 (Adverse) |
| AOI_Newborn | D12 | -0,77 | 0.051 | 6 | r<0 (Adverse) |
| AOI_Newborn | PSC | -0,89 | 0.008* | 6 | r<0 (Adverse) |
| AOI_Total | D1 | -0,77 | 0.051 | 6 | r<0 (Adverse) |
| AOI_Total | D2 | -1 | 0.001* | 6 | r<0 (Adverse) |
| AOI_Total | D3 | -0,43 | 0.178 | 6 | r<0 (Adverse) |
| AOI_Total | D4 | -0,43 | 0.178 | 6 | r<0 (Adverse) |
| AOI_Total | D5 | -0,54 | 0.149 | 6 | r<0 (Adverse) |
| AOI_Total | D6 | -0,83 | 0.017* | 6 | r<0 (Adverse) |
| AOI_Total | D7 | -0,71 | 0.068 | 6 | r<0 (Adverse) |
| AOI_Total | D8 | -0,77 | 0.051 | 6 | r<0 (Adverse) |
| AOI_Total | D9 | -0,43 | 0.178 | 6 | r<0 (Adverse) |
| AOI_Total | D10 | -0,94 | 0.008* | 6 | r<0 (Adverse) |
| AOI_Total | D11 | -0,89 | 0.008* | 6 | r<0 (Adverse) |
| AOI_Total | D12 | -0,66 | 0.068 | 6 | r<0 (Adverse) |
| AOI_Total | PSC | -0,94 | 0.008* | 6 | r<0 (Adverse) |
| WAOS_Total | D1 | -0,26 | 0.282 | 6 | r<0 (Adverse) |
| WAOS_Total | D2 | -0,77 | 0.051 | 6 | r<0 (Adverse) |
| WAOS_Total | D3 | -0,03 | 0.460 | 6 | r<0 (Adverse) |
| WAOS_Total | D4 | -0,43 | 0.178 | 6 | r<0 (Adverse) |
| WAOS_Total | D5 | -0,43 | 0.178 | 6 | r<0 (Adverse) |
| WAOS_Total | D6 | -0,49 | 0.149 | 6 | r<0 (Adverse) |
| WAOS_Total | D7 | -0,2 | 0.357 | 6 | r<0 (Adverse) |
| WAOS_Total | D8 | -0,31 | 0.282 | 6 | r<0 (Adverse) |
| WAOS_Total | D9 | -0,87 | 0.017* | 6 | r<0 (Adverse) |
| WAOS_Total | D10 | -0,66 | 0.068 | 6 | r<0 (Adverse) |
| WAOS_Total | D11 | -0,49 | 0.149 | 6 | r<0 (Adverse) |
| WAOS_Total | D12 | -0,37 | 0.249 | 6 | r<0 (Adverse) |
| WAOS_Total | PSC | -0,66 | 0.068 | 6 | r<0 (Adverse) |
| SI_Total | D1 | 0,09 | 0.599 | 6 | r<0 (Adverse) |
| SI_Total | D2 | -0,49 | 0.149 | 6 | r<0 (Adverse) |
| SI_Total | D3 | 0,09 | 0.599 | 6 | r<0 (Adverse) |
| SI_Total | D4 | -0,43 | 0.178 | 6 | r<0 (Adverse) |
| SI_Total | D5 | -0,49 | 0.149 | 6 | r<0 (Adverse) |
| SI_Total | D6 | -0,37 | 0.249 | 6 | r<0 (Adverse) |
| SI_Total | D7 | 0,03 | 0.540 | 6 | r<0 (Adverse) |
| SI_Total | D8 | -0,14 | 0.401 | 6 | r<0 (Adverse) |
| SI_Total | D9 | -0,99 | 0.001 | 6 | r<0 (Adverse) |
| SI_Total | D10 | -0,31 | 0.282 | 6 | r<0 (Adverse) |
| SI_Total | D11 | -0,09 | 0.401 | 6 | r<0 (Adverse) |
| SI_Total | D12 | 0,09 | 0.599 | 6 | r<0 (Adverse) |
| SI_Total | PSC | -0,31 | 0.282 | 6 | r<0 (Adverse) |

Note: * indicates p<0.05, one-sided exact; EP ρ>0; adverse ρ<0); no multiplicity adjustment.
