# Supplementary material for: Association between patient safety culture, adverse events, and essential practices during childbirth in six Brazilian maternity hospitals
Source: Int J Gynaecol Obstet. 2026 Mar 12;174(3):1316–27. doi: 10.1002/ijgo.70890 (PMC13505257; doi:10.1002/ijgo.70890)
Supplement: Supplementary file 2 [file IJGO-174-1316-s002.docx]

**Supplementary Table S2.**

**Respondent Professional Categories by Hospital (H1–H6) and Overall**

| **Professional category** | **H1** | **H2** | **H3** | **H4** | **H5** | **H6** | **Total** |
| --- | --- | --- | --- | --- | --- | --- | --- |
| **Nurse** | 14,3% | 18,9% | 6,3% | 13,1% | 11,4% | 33,3% | 14,1% |
| **Nursing assistant/technician** | 28,6% | 24,6% | 0,0% | 29,5% | 40,0% | 26,7% | 29,2% |
| **Staff physician** | 5,6% | 6,6% | 0,0% | 6,8% | 7,1% | 13,3% | 6,4% |
| **Resident physician** | 0,8% | 0,8% | 59,4% | 0,0% | 0,0% | 0,0% | 3,1% |
| **Pharmacist** | 5,6% | 3,3% | 3,1% | 2,8% | 4,3% | 0,0% | 3,6% |
| **Administrative assistant/secretary** | 4,0% | 9,0% | 0,0% | 0,8% | 5,0% | 0,0% | 3,6% |
| **Support technicians** | 4,8% | 4,1% | 0,0% | 2,4% | 5,0% | 0,0% | 3,5% |
| **Physiotherapist** | 4,0% | 4,1% | 3,1% | 2,4% | 1,4% | 0,0% | 2,8% |
| **Administration/Management** | 4,0% | 1,6% | 0,0% | 0,8% | 0,0% | 6,7% | 1,5% |
| **Social worker** | 0,0% | 0,8% | 0,0% | 1,6% | 2,1% | 0,0% | 1,2% |
| **Nutritionist** | 1,6% | 1,6% | 6,3% | 0,8% | 0,0% | 0,0% | 1,2% |
| **Psychologist** | 0,8% | 0,8% | 3,1% | 0,4% | 0,0% | 0,0% | 0,6% |
| **Dentist** | 0,0% | 1,6% | 0,0% | 0,0% | 0,7% | 0,0% | 0,4% |
| **Other** | 8,7% | 2,5% | 3,1% | 19,9% | 7,1% | 6,7% | 11,1% |
| **Not reported** | 17,5% | 19,7% | 15,6% | 18,7% | 15,7% | 13,3% | 17,8% |
| **Total** | 100,0% | 100,0% | 100,0% | 100,0% | 100,0% | 100,0% | 100,0% |
