# Supplementary material for: Association between patient safety culture, adverse events, and essential practices during childbirth in six Brazilian maternity hospitals
Source: Int J Gynaecol Obstet. 2026 Mar 12;174(3):1316–27. doi: 10.1002/ijgo.70890 (PMC13505257; doi:10.1002/ijgo.70890)
Supplement: Supplementary file 3 [file IJGO-174-1316-s003.docx]

**Supplementary Table S1.**

**Adverse Event Composite Measures—Definitions and Scoring (AOI, WAOS, SI)**

| **AE Indicators** | **Concept** | **Additional details** |
| --- | --- | --- |
| Adverse Outcome Index (AOI) | It is the percent of deliveries with  one or more adverse events. | The medical records of mother and newborn are counted only once if an adverse event is registered on either of them. |
| Weighted Adverse Outcome Score (WAOS) | It describes the adverse  event score per delivery. It is the sum of the points  assigned to cases with adverse outcomes divided by the number of deliveries. | Scores per AE:  Maternal death: **750**  Intrapartum or neonatal death > 2,500g*: **400**  Uterine rupture: **100**  Maternal admission to ICU: **65**  Birth trauma: **60**  Return to OR / labor & delivery: **40**  Admission to NICU >2,500 g & for > 24 hours: **35**  APGAR < 7 at 5 minutes: **25**  Blood transfusion: **20**  3º- or 4º-perineal tear: **5** |
| Severity Index (SI) | It describes the severity of the outcomes. It is the sum of the adverse outcome scores divided by the number of deliveries with an identified adverse  outcome. |  |
| Mann *et al.*, 2006.* For this study, the score of Intrapartum or neonatal death was calculated for any fetal weight. | | |
